# Detailed Revision Risk Analysis after Single- vs. Two-Stage Revision Total Knee Arthroplasty in Periprosthetic Joint Infection: A Retrospective Tertiary Center Analysis

**DOI:** 10.3390/antibiotics10101177

**Published:** 2021-09-27

**Authors:** Lars-Rene Tuecking, Julia Silligmann, Peter Savov, Mohamed Omar, Henning Windhagen, Max Ettinger

**Affiliations:** 1 Department of Orthopaedic Surgery, Hannover Medical School, Diakovere Annastift, Anna-von-Borries-Str. 1-7, 30625 Hannover, Germany; lars-rene.tuecking@diakovere.de (L.-R.T.); julia.silligmann@diakovere.de (J.S.); peter.savov@diakovere.de (P.S.); Henning.Windhagen@diakovere.de (H.W.); 2 Department of Trauma Surgery, Hannover Medical School, Carl-Neuberg-Strasse 1, 30625 Hanover, Germany; omar.mohamed@mh-hannover.de

**Keywords:** periprosthetic joint infection, PJI, single-stage revision TKA, two-stage revision TKA, revision risk, rTKA

## Abstract

Periprosthetic joint infection (PJI) remains one of the most common causes of revision knee arthroplasty. Controversy continues to surround the proper operative technique of PJI in knee arthroplasty with single- or two-stage replacement. Significant variations are seen in the eradication rates of PJI and in implant survival rates. This detailed retrospective analysis of a single tertiary center is intended to provide further data and insight comparing single- and two-stage revision surgery. A retrospective analysis of all revision total knee arthroplasty (TKA) surgeries from 2013 to 2019 was performed and screened with respect to single- or two-stage TKA revisions. Single- and two-stage revisions were analyzed with regard to implant survival, revision rate, microbiological spectrum, and other typical demographic characteristics. A total of 63 patients were included, with 15 patients undergoing single-stage revision and 48 patients undergoing two-stage revision. The mean follow-up time was 40.7 to 43.7 months. Statistically, no difference was found between both groups in overall survival (54.4% vs. 70.1%, *p* = 0.68) and implant survival with respect to reinfection (71.4% vs. 82.4%, *p* = 0.48). Further, high reinfection rates were found for patients with difficult-to-treat organisms and low- to semi-constrained implant types, in comparison to constrained implant types. A statistically comparable revision rate for recurrence of infection could be shown for both groups, although a tendency to higher reinfection rate for single-stage change was evident. The revision rate in this single-center study was comparably high, which could be caused by the high comorbidity and high proportion of difficult-to-treat bacteria in patients at a tertiary center. In this patient population, the expectation of implant survival should be critically discussed with patients.

## 1. Introduction

Periprosthetic joint infection (PJI) in knee arthroplasty is one of the major causes of revision surgery [1,2]. In 14.5% to 25.2% of cases after primary total knee arthroplasty (TKA), revisions are caused by PJI. Further, an increased risk of failure due to PJI is found in patients undergoing revision total knee arthroplasty (rTKA) [3]. In a single-center study of 566 rTKA, 46% of re-revisions were caused by PJI [3]. Keeping in mind that the numbers for TKA are steadily increasing [4], the number of PJIs can also be expected to increase significantly, if infection rates are remaining constant. Despite established and studied treatment strategies, PJI shows a high occurrence rate of reinfection with 19% to 23% [5,6]. The diagnosis and treatment of PJI often have serious consequences for patients with significant morbidity and mortality [7,8] and also pose serious challenges to health care systems [4].

While early onset infections are usually treated with open debridement, replacement of the tibial insert, and targeted antibiotic therapy, late-onset infections are treated with component replacement, either in a one- or two-stage procedure. For many years, two-stage replacement of knee arthroplasty for infections was the gold standard [9]. To overcome the morbidity of two-stage procedures, septic single-stage arthroplasty was introduced [10]. Two-stage revision involves removal of the prosthesis, installation of static or mobile antibiotic-loaded cement spacers, leaving the spacers in place for approximately 6 weeks with concurrent antibiotic therapy, followed by reimplantation of the prostheses and renewed postoperative antibiotic therapy [9]. The single-stage procedure involves radical soft tissue debridement and osseous debridement with direct reimplantation of the prosthesis with the use of antibiotic-loaded cement and postoperative antibiotic therapy [9].

Single-stage revision offers benefits in terms of health economic costs, hospitalization and length of stay, and improved morbidity, as well as patient satisfaction [11,12]. However, concerns still remain that higher reinfection rates may persist after single-stage procedures [13]. Reinfection rates in between different studies of single- or two-stage revision TKA procedures vary significantly. Reported reinfection rates vary from 7.6% to 38.25% [14], regardless of the technique used. Lately, several studies report comparable outcomes and reinfection rates for direct comparison of single- vs. two-stage revision arthroplasty [15,16,17]. Nevertheless, there are still uncertainties, for example, regarding the influence of patient selection and indication criteria for single-stage TKA revision [17,18]. Furthermore, studies show large variations in reinfection rates [14] so that in addition to large, methodologically highly qualitative studies, data from retrospective studies with an exact evaluation of the patients’ demographic data are still needed.

For this reason, this single tertiary center analysis examined the midterm results after single- and two-stage revision to add valuable data on survival rates and reinfection rates, in comparison to both techniques. Detailed analysis on patients’ demographics and risk factors was carried out.

## 2. Results

### 2.1. Group Sizes and Patient Inclusion

**Figure 1:**
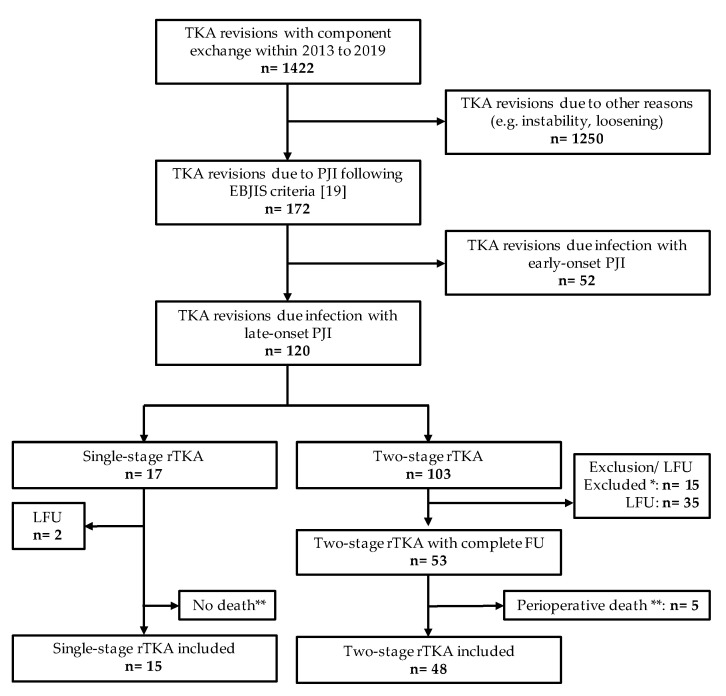
Study flow diagram: TKA, total knee arthroplasty; rTKA, revision TKA; PJI, periprosthetic joint infection; EBJIS, European Bone and Joint Infection Society; LFU, lost to follow-up; FU, follow-up; *, patients did not meet inclusion criteria due to incomplete data for PJI evaluation, e.g., inadequate number of intraoperative tissue samples for microbiological analysis; **, died in the course of implant removal or reimplantation procedure and analyzed separately.

A total of 68 cases were included in this single-center retrospective cohort study comparing single- and two-stage TKA revision, whereas only 63 cases were available for implant survival and reinfection risk analysis. The in-clinic database consisted of 1422 TKA revisions, with component exchange from 01/2013 to 12/2019, whereas of these 120 patients showed a late-onset PJI following the EBJIS criteria (Figure 1). A total of 50 cases needed to be excluded in the two-stage group due to the loss of follow-up, i.e., we were unable to contact patients or incomplete microbiological or pathological data necessary for PJI evaluation following EBJIS criteria was not available. A total of two cases were unable to be contacted in the single-stage revision group.

Within the course of the two-stage TKA revisions, five patients died (mortality rate: 9.4%), whereas none of the patients in the single-stage group died within the perioperative course or in relation to the operation. Of those five cases in the two-stage group, two patients died in the period between prosthesis explantation and reimplantation. Three of the five patients died after reimplantation in the direct postoperative course. Other deaths were included in the Kaplan–Meier analysis. However, only one additional death was observed in the two-stage group, deceased 10 months postoperatively.

### 2.2. Demographic Data

Demographic data of both groups are summarized in Table 1. Mean follow-up time was comparable between single-stage revision and two-stage revision groups (47.3 ± 19.2 months, 40.7 ± 23.1 months; *p* = 0.982). Age, body mass index (BMI), American Society of Anesthesiologists physical status score (ASA), and microbiological organism characteristics (difficult-to-treat organisms (DTT)) showed no differences between the groups (Table 1). The distribution of gender and used implant types showed differences between the groups (Table 1). Although low constraint prostheses (cruciate-retaining (CR)/posterior stabilized (PS), and semi-constrained) were mainly used in the single-stage group (78.5%), the proportion of constraint prostheses predominated in the two-stage group (56.3%, *p* = 0.048). In the two-stage group, mobile spacers (77.1%) were predominantly used.

The distribution of microbiological organisms between the groups is summarized in Table 2. The most common organisms were coagulase-negative staphylococci (CNS) in both groups, with 33.3% in the single-staged revision group and 27.1% in the two-stage revision group, respectively. A high amount of *Staphylococcus* spp. (45.5%) found in the two-stage revision group showed difficult-to-treat characteristics, whereas only 14.3% of the *Staphylococcus* spp. in the single-stage revision group were DTT. Overall, the proportion of DTT organisms was very high in both groups (26.4%, 37.5%, respectively; *p* = 0.544).

### 2.3. Survival Rate Analysis

**Figure 2:**
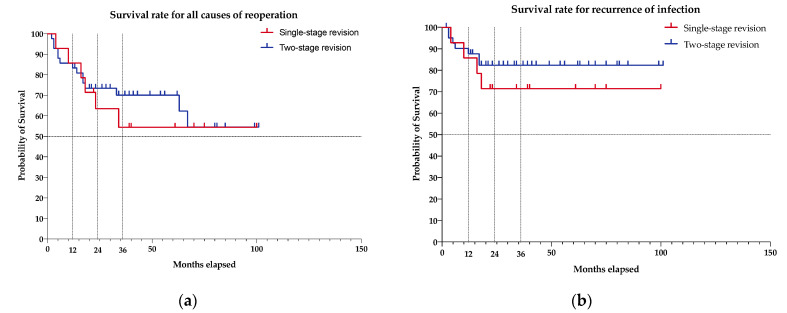
Kaplan–Meier survival analysis following single- and two-stage total knee arthroplasty revision after periprosthetic joint infection: (**a**) survival rate for all causes for re-revision; (**b**) survival rate for recurrence of infection.

The mean survival did not differ in between groups due to all causes of revision (*p* = 0.684) and due to recurrence of infection (*p* = 0.419), especially in the first 18 months (Figure 2). Despite no statistical difference, the single-stage group showed higher revision rates, especially with regard to infection-related revision (Figure 2, Table 3). The overall Kaplan–Meier survival rate (Figure 2) was 85.7% (95% Ci 53.9 to 96.2) at 12 months for single-stage revisions and 83.3% (95% Ci 68.2 to 91.7) for two-stage revisions, 63.5% (95% Ci 33.1 to 83.0) vs. 73.4% (95% Ci 57.1 to 84.3) at 24 months, and 54.4% (95% Ci 24.8 to 76.7) vs. 70.1% (95% Ci 53.2 to 81.9) at 36 months. Regarding the recurrence of infection, the Kaplan–Meier survival rate was 85.7% (95% Ci 53.9 to 96.2) for single-stage revisions and 87.7% (95% Ci 72.9 to 94.7) for two-stage revisions at 12 months, 71.4% (95% Ci 40.6 to 88.2) and 82.4% (95% Ci 66.5 to 91.2) at 24 and 36 months.

At final follow-up, a total of 7/15 cases (53.3%) had to be revised in the single-stage group and 15/48 cases (31.3%) in the two-stage group, whereas 4/15 cases (26.7%, single-stage) and 7/48 (14.6%, two-stage) were revised due to reinfection. Recurrence of infection with the same germ was seen in 2/4 cases in the single-stage group (50%) and in 3/7 cases in the two-stage group (42.9%). No germ but other positive PJI criteria were found in 2/4 cases in the single-stage group (50%) and in 3/7 cases in the two-stage group (42.9%). Reinfection was found in 2/7 cases in the two-stage group (28.6%). The time between index operation and overall re-revision and revision due to infection was comparable between both groups (Table 3, *p* = 0.331, *p* = 0.497, respectively).

### 2.4. Influence of Variables on Reinfection Rate

Statistical analysis of the influence of variables on the reinfection rate (gender, ASA score, McPherson score, number of preoperations, implant type of reimplantation, spacer type, or organism characteristics) did not show any significant risk factor (all *p* > 0.05). However, it was striking that especially cases with a low constraint level implant restoration (CR/PS or semi-constrained) were mainly responsible for reinfections in both groups (100.0% of single-stage revisions, 85.7% of two-stage revisions, Table 4). Furthermore, reinfections in the two-stage group were only found in cases restored with a mobile spacer, while all cases (n = 11) with static spacers showed no revision due to reinfection. The reinfection rate in the two-stage group was also very high in cases with DTT organisms. Here, 71.4% of all reinfections in this group were found in cases with DTT organisms.

## 3. Discussion

The main result of this study was a comparable survival rate after one-stage and two-stage revision knee arthroplasty in the presence of periprosthetic infection. Furthermore, the revision rate for recurrence of infection was statistically comparable, with a tendency toward a higher r-infection rate after single-stage replacement. There was also an association of higher reinfection rates with low- and semi-constrained prostheses, compared to constrained prostheses.

Recently published studies mainly report comparable eradications rates and functional outcomes for single-stage (87%) and two-stage revision TKA (85%) [20]. Therefore, single-stage revision TKA became an increasingly used method due to possibly reducing surgical procedures, antibiotic treatment, and costs [20]. Accordingly, this cohort analysis also did not show any statistical differences between both groups in terms of overall implant survival or infection-free implant survival. Nevertheless, a tendency to higher reinfection rates was found in the single-stage group. Despite the comparable revision rates in both groups of this study, comparatively high infection-related revision rates were seen in this cohort study. A recent review of 29 studies by Lazic et al. [21] showed a mean eradication rate of 87 ± 8.8% (single-stage revision) and 83 ± 11.7% (two-stage revision) in the treatment of PJI in TKA. Our cohort study showed significantly higher revision rates in this comparison, with implant survival in terms of reinfection of 71.4% (single-stage revision) and 82.4% (two-stage revision) at 2 years. While a detailed analysis of patient demographics is limited in a review, our cohort study shows a high proportion of difficult-to-treat organisms (approximately one-third of patients in both groups), as the cause of PJI, and high patient morbidity, as measured by the McPherson score. Frequently, the patient population in tertiary centers for orthopedic surgery is associated with increased morbidity and multiple prior surgeries so that presentation to a tertiary center usually occurs only after multiple prior surgeries. Accordingly, the proportion of multi-morbid patients is increased. Wimmer et al. [19] have already shown that PJIs with difficult-to-treat organisms occur mainly in patients with multiple comorbidities and thus increase the risk of revision or reinfection. Furthermore, there are also studies that specifically investigate implant survival after PJI revision with a complicated or resistant organism (Table 5).

In these studies, multidrug-resistant or difficult-to-treat organisms show significantly reduced implant survival rates (Table 5) [19,22,23,24,25,26]. In their cohort study, Wimmer et al. showed an implant survival rate of 68.9% in two-stage TKA revisions in patients with DTT organisms [19]. In comparison, the two-stage cohort of the current study showed an infection-free implant survival of 82.4% after 2 years. However, it should be noted that only one-third of the patients in this cohort showed DTT organism in microbiological analysis. Another reason for the reduced survival rate of the implants in the current study could be the comparably high comorbidity scores (Mc Pherson score) of the included patients. For example, more than 50% of the patients with reinfection in the two-stage group presented with a local extremity grade of III and approximately 43% with a host grade of C (Table 4). Wimmer et al. found a significant association between reinfection risk and McPherson score [19]. It should be noted that in the presence of high patient comorbidity or difficult-to-treat organism, a reduction in the success rate after one- or two-stage change should be assumed and this should definitely be communicated with the patient in terms of expectations. The results of revision surgery in a study population with high-risk patients and complicated microbiological organism structure must necessarily be studied and reported separately from revision cases with low-risk patients and multi-sensitive organism structure.

Another factor in the present retrospective analysis was the increased reinfection rate in patients with low- (CR/PS) and semi-constraint (= condylar constrained knee, CCK) prostheses. In the single-stage group, all reinfections were treated with a CR/PS or CCK prosthesis, and in the two-stage group, 85.7% of the patients were treated with these implant systems. One reason for the higher reinfection rate may be the reduced radicality of the revision surgery since a substantial bone stock and soft tissue envelop (e.g., collateral ligaments) must be functionally present during reimplantation of CR/PS or CCK prostheses. In the context of a revision with constrained prostheses, a considerably more radical revision is possible and, in some cases, also necessary. This may promote infection eradication. Appropriately, a recent study by Ohlmeier et al. [27] showed an eradication rate of PJI of 90% 6 years postoperatively after single-stage TKA revision with the use of rotation hinge prostheses. Nevertheless, this study showed an increased overall revision rate after rotating hinge prostheses with an overall survival rate of 75% at 6 years [27]. In contrast, a retrospective study of 132 patients with single-stage rotation and CCK prostheses showed an eradication rate of 91% and an overall survival rate of 82.7% at 5 years [28]. Overall, however, it should be noted that the data available for the comparison of different implant systems in PJI treatment are still very limited. Further studies on the direct comparison of CCK and hinged prostheses should follow. In particular, it should be investigated which implant survival is shown after low- or semi-constrained implant treatment in patients with evidence of DTT organism or poor comorbidity scores.

As other studies have shown [29,30], patient selection for single-stage revisions remains critical. Polymicrobial infections are a major factor. In our institution, polymicrobial infections are not treated by single-stage revision if they are already known preoperatively. Although our results cannot prove this, the knowledge about the germ and the resistance status of this germ is very helpful in our experience. This might be essential, especially in cases with concurrent high risk factors (poor bone stock or poor soft tissue status). Nevertheless, negative preoperative cultures in single-stage revisions do not necessarily have to be an exclusion criterion, as recently shown by van den Kieboom et al. [15]. Moreover, the possibility of one-stage revision should be considered as an option despite the presence of risk factors regarding reinfection, especially in patients with increased perioperative mortality risk. This has to be considered, especially considering the mortality rate, also indicated in our study, after two-stage revision. Five patients (9.4%) died within the course of the two-stage procedure or directly after reimplantation, whereas no perioperative death was observed in the single-stage group. As mentioned before, these results also have to be evaluated with limitations, since the number of cases in the single-stage group is significantly lower. Nevertheless, this is another indication of a significant mortality risk in the direct course of two-stage revisions. In this context, Pelt et al. found a mortality rate of 8.6% in the interval between explantation and reimplantation in their analysis of two-stage TKA revision procedures [31]. Lum et al. described an overall mortality rate after two-stage TKA revision of 14.4%, with a mean follow-up of 3.8 years [4].

This study shows certain limitations. The main limitation is the limited number of cases and thus significantly reduced statistical power. Despite numerous clear abnormalities with regard to reinfections, these were not statistically significant, which could be due to the small number of cases and correspondingly significantly reduced power. Despite statistically non-significant differences, however, this detailed analysis was able to show individual abnormalities with regard to the risk of reinfection and to illustrate the possibly significantly higher revision rates in high-risk patients or complicated organism structures. Furthermore, the demographic data of the groups showed differences in gender distribution, implant systems, and McPherson local extremity scores, which might have influenced the results comparing both groups. In addition, there was also significant heterogeneity of patients within the groups, which may equally limit the validity of the present results. It should also be noted that only one polymicrobial infection was included in the single-stage group. For this reason, the results regarding polymicrobial infections must be evaluated with this limitation. In this context, however, it must be made clear that a polymicrobial infection, which was already known preoperatively, was considered an exclusion criterion for a single-stage procedure in this study.

## 4. Materials and Methods

A retrospective cohort analysis was undertaken of all rTKA performed in a single university center of orthopedic surgery (high-volume arthroplasty center) from 2013 to 2019. All cases of revision TKA were screened for the usual criteria for late-onset PJI (criteria of European Bone and Joint Infection Society (EBJIS) [29]) and then classified into two cohorts: single-stage or two-stage revision procedures.

The primary outcome of this study was implant survivorship defined by the cumulative incidence of revision or revision due to reinfection in comparison to single- and two-stage TKA revision. A revision was considered to be any procedure that involved the removal of an implant (polyethylene insert, patellar component, femur, or tibia). Included patients were censored at death or last patient contact at the time course of study data collection. The secondary outcome was variable analysis on infection-free survivorship including gender, ASA score, McPherson score, number of preoperations, implant type, spacer type, and difficult-to-treat organism characteristics.

Survival of prosthesis and revision risk was analyzed by contacting patients or examination during an outpatient consultation. Patients were investigated regarding further revision surgery (time of surgery, reason, location). In unclear cases, further treating physicians or clinical centers were contacted if revision surgeries were performed outside our hospital center. The minimal follow-up time was 18 months. Exclusion criteria were tumor or trauma-related surgeries, lost to follow-up, incomplete diagnostic data (e.g., less than three microbiological samples missing or incomplete pathological examination), and declined participation. Death within the perioperative course of single- or two-stage revision procedures were analyzed separately to allow a pure follow-up analysis regarding implant survival and reinfection risk.

The in-clinic database was analyzed for age, BMI, sex, ASA score/Musculoskeletal Infection Society score (MSIS), time of surgery, time to revision, reasons for revision, implants previous to revision, microbiological specimen, and antibiotic drug usage.

Diagnosis of PJI was conducted using criteria of EBJIS, as described in the review of Izakovicova et al. [29]. PJI was diagnosed if one or more of the following criteria were found: (1) macroscopic infection signs (purulence or sinus tract), (2) leukocyte cell count in synovial fluid (>2000 leukocytes per µL or >70% granulocytes (polymorphonuclear leukocytes)), (3) periprosthetic tissue histology following the criteria of Krenn and Morawietz (≥23 granulocytes per 10 high-power fields) [32], and (4) positive microbiological tissue samples (> 2 positive samples of minimum > 3 samples) or positive microbiological synovial fluid analysis. Therefore, PJI might be diagnosed due to the mentioned criteria with negative cultures of pre- or perioperative samples. The definition of difficult-to-treat microbiological organism was based on the definition by Wimmer et al. [19].

The institutional absolute criteria against single-stage revision procedure were concurrent sepsis, impaired immune status (immunocompromise medication, immunocompromise oncologic diseases), and preoperative known polymicrobial infection status. Relative criteria against single-stage revision were preoperative existing massive bone defect (AORI Type III) or weak soft tissue status (local grade III), with risk of postoperative necrosis (e.g., due to strong subcutaneous adhesions, thin skin covering). All patients not matching for single-stage TKA revision were chosen for the two-stage revision procedure. The surgical technique used in the one- and two-stage procedures is similar to the technique described by Zahar et al. [9]. In both procedures, the lowest possible constraint level of the prostheses was selected depending on the bone and soft tissue loss and, if necessary, supplemented with cones, augments, and sleeves.

Antibiotic-loaded cement was used in both groups for reimplantation of the prostheses if it matched the antibiogram of the detected organisms, and in the other cases, with vancomycin and gentamycin. In the single-stage procedure, i.v. antibiotics were given postoperatively for 2 weeks, followed by oral antibiotics for 10 weeks. In our institution, a total duration of antibiotic therapy of 12 weeks is chosen for the one-stage change. The effectiveness of this duration time has already been proven [33] and is also based on the EJBS/Proimplant Foundation recommendations [29]. Antibiotic therapy was started intraoperatively directly after tissue sampling. In the two-stage procedure, i.v. antibiotics were administered for 2 weeks after implant removal (likewise intraoperative start of i.v. therapy), followed by oral antibiotics for 4 weeks. As also recommended by the Pro-Implant Foundation [29], reimplantation after 6 weeks was followed by i.v. antibiotics for 2 weeks and another oral antibiotic therapy for 6 weeks. No interruption of antibiotics was performed, and no aspiration of the joint was performed preoperatively before reimplantation. Initial intravenous antibiotic therapy was started with vancomycin and clindamycin and was then de-escalated as soon as pathogens were known or adapted to the resistance testing in collaboration with microbiologists. Adapted to the resistance situation, if possible, dual antibiotic therapy for i.v. and oral administration was given. Rifampicin, effective against biofilm, was added to the treatment in cases of infection with *Staphylococcus spp.* or Gram-positive Anaerobes as soon as skin wound showed no drainage and resistogram was suitable. In those cases, oral antibiotic therapy included rifampicin in combination with fluocinolones or tetracyclines (ampicillin in cases of Gram-positive Anaerobes). Further therapy for the other germs was determined individually with the microbiologists for each patient.

Statistical analysis was performed using GraphPad Prism V9.0 (USA) and Microsoft Excel. Kaplan–Meier curve analysis with revision for any reason and revision for reinfection for endpoints were created using 95% confidence intervals. Nominal variables were compared using Fisher’s exact test, ordinal variables were compared using a chi-square test for trend, metric variables were tested on normality with Shapiro–Wilk test and then compared with unpaired two-tailed *t*-test or Mann–Whitney U test depending on the results of Shapiro–Wilk tests. Statistical significance level was set to *p* value < 0.05 for all statistical tests.

## 5. Conclusions

This retrospective analysis showed comparable revision rates between one- and two-stage TKA revision with PJI present. Reinfection rates were also statistically comparable but tended to be higher in the one-stage group. In addition, there was evidence of higher reinfection rates in low- and semi-constrained prostheses after PJI.

## Figures and Tables

**Table 1:** Group-specific demographic data.

Demographic Variables		Single-Stage	Two-Stage	*p* Value
Group size	n	15	48	
Age (years)	mean (±SD)	65.0 (±10.2)	69.3 (±11.1)	0.315
	min	49.0	51.0	
	max	84.0	93.0	
BMI (kg/ m^2^)	mean (±SD) min	30.1 (±5.9) 20.2	29.9 (±7.2) 19.6	0.725
	max	42.6	46.3	
Gender	female	11 (73.3%)	20 (41.7%)	0.041 *
ASA score	I	2 (13.3%)	1 (2.1%)	0.377
	II	9 (60.0%)	33 (68.8%)	
	III	4 (26.7%)	14 (29.2%)	
	IV			
Mc Pherson score	Host grade A	7 (46.7%)	8 (16.7%)	0.150
	B	4 (26.7%)	27 (56.3%)	
	C	4 (26.7%)	13 (27.1%)	
	Local grade I	4 (26.7%)	2 (4.2%)	0.015 *
	II	7 (46.7%)	18 (37.5%)	
	III	4 (26.7%)	28 (58.3%)	
Number of preoperations	mean (± SD)	2.1 (±1.2)	3.3 (±2.3)	0.081
Follow up (months)	mean (± SD)	47.3 (±19.2)	40.7 (±23.1)	0.982
	min	22.0	18.0	
	max	75.0	92.0	
Implants after revision	CR/ PS	3 (20.0%)	6 (12.5%)	0.048*
	semi constrained	8 (53.3%)	13 (27.1%)	
	constrained	4 (26.7%)	27 (56.3%)	
	DFR		1 (2.1%)	
	arthrodesis		1 (2.1%)	
Spacer type	mobile		37 (77.1%)	- - -
	static		11 (22.9%)	
Organism characteristics	DTT	4 (26.4%)	18 (37.5%)	0.544
	non DTT	11 (73.3%)	30 (62.5%)	

SD, standard deviation; ASA, American Society of Anesthesiologists score; MSIS, Musculoskeletal Infection Society score; CR, cruciate retaining; PS, posterior stabilized; DFR, distal femoral replacement; DTT, difficult to treat [19]; * statistically significant.

**Table 2:** Distribution of microbiological organisms.

Microbiological Organism	Resistance	Single-Stage	Two-Stage
**Gram positive**			
Coagulase-negative staphylococcus		5 (33.3%)	13 (27.1%)
*of those:*	*Methicillin/Clindamycin*		*2 (4.2%)*
	*Rifampicin*		*2 (4.2%)*
Staphylococcus aureus			5 (10.4%)
*of those:*	*Methicillin*		*1 (2.1%)*
Divers Staphylococcus spp.		2 (13.3)	4 (8.33%)
Streptococcus spp.			2 (4.2%)
Bacillus spp.			1 (2.1%)
Microccocus luteus			1 (2.1%)
Enterococcus faecalis		1 (6.7%)	3 (6.3%)
Propriobacterium acnes		1 (6.7%)	
			
**Fungi**			
Candida spp.		1 (6.7%)	1 (2.1%)
			
**Others**			
Polymicrobial		1 (6.7%)	6 (12.5%)
No growth		4 (26.7%)	11 (22.9%)

**Table 3:** Revision rate analysis at final follow-up.

	Cause of Revision		Single-Stage	Two-Stage	*p* Value **
Revision rate					
	**Overall**	**n (% of all)**	**7 (53.3%)**	**15 (31.3%)**	**0.684**
				
	**Infection**	n (% revision/% all)	**4 (57.1%/26.7%)**	**7 (46.7%/14.6%)**	**0.419**
	Loosening *		2 (28.6%/13.3%)	2 (13.3%/4.2%)	- - -
	Fracture			2 (13.3%/4.2%)	- - -
	Instability		1 (14.3%/6.7%)	1 (6.7%/2.1%)	- - -
	Unknown			3 (20.0%/6.3%)	- - -
Time to revision	Overall	Months (± SD)	15.0 (± 5.5)	15.4 (± 16.3)	0.331
	Infection	Months (± SD)	12.0 (± 10.1)	9.3 (± 5.7)	0.497

SD, standard deviation; *, aseptic loosening; **, Kaplan–Meier analysis.

**Table 4:** Variable analysis on revision rate.

Variables		Single-Stage			Two-Stage		
		Overall	Infection	*p* Value *	Overall	Infection	*p* Value *
Gender	female	5 (71.4%)	4 (100.0%)	0.52	6 (40.0%)	1 (14.3%)	0.21
	male	2 (28.6%)			9 (60.0%)	6 (85.7%)	
ASA score	I	1 (14.3%)	1 (25.0%)	0.61			0.11
	II	4 (57.1%)	2 (50.0%)		13 (86.7%)	7 (100.0%)	
	III	2 (28.6%)	1 (25.0%)		2 (13.3%)		
	IV						
	Host grade						
Mc Pherson score	A	4 (57.1%)	3 (75.0%)	0.12	1 (6.7%)		0.15
	B	2 (28.6%)	1 (25.0%)		8 (53.3%)	4 (57.1%)	
	C	1 (14.3%)			6 (40.0%)	3 (42.9%)	
	Local grade						
	I	2 (28.6%)	1 (25.0%)	>0.99			0.88
	II	3 (42.9%)	2 (50.0%)		8 (53.3%)	3 (42.9%)	
	III	2 (28.6%)	1 (25.0%)		7 (46.7%)	4 (57.1%)	
Number of preoperations	Mean (± SD)	1.6 (±0.7)	1.3 (± 0.4)	0.13	2.9 (±1.6)	2.4 (±1.0)	0.41
Implants after revision	CR/ PS	2 (28.6%)	1 (25.0%)	0.52	2 (13.3%)	2 (28.6%)	0.10
	Semi-constrained	3 (42.9%)	3 (75.0%)		6 (40.0%)	4 (57.1%)	
	Constrained	2 (28.6%)			7 (46.7%)	1 (14.3%)	
Spacer type	Mobile				13 (86.7%)	7 (100.0%)	0.18
	Static				2 (13.3%)		
Organism characteristics	DTT	2 (28.6%)		0.52	9 (60.0%)	5 (71.4%)	0.09
	non DTT	5 (71.4%)	4 (100.0%)		6 (40.0%)	2 (28.6%)	

ASA, American Society of Anesthesiologists score; MSIS, Musculoskeletal Infection Society score; CR, cruciate retaining; PS, posterior stabilized; DFR, distal femoral replacement; DTT, difficult to treat [19]; * chi-square test for trend.

**Table 5:** Implant survival rates after PJIs associated with difficult-to-treat organisms.

Authors	Year	Journal	Patients	Microbiological Organism	Follow-Up	Procedures	Implant Survival
Wimmer et al. [19]	2020	J Diagmicrobio	45	DTT	2 yr	Two-stage	68.9%
Thompson et al. [22]	2019	JBJI	55	Enterococcus	5 yr	Single-/Two-stage	67–80%
Kheir et al. [23]	2017	J Arthroplasty	87	Enterococcus	4 yr	DAIR/Single-/Two-stage	39.4%/45.5%/62.8%
Vasso et al. [24]	2016	KSSTA	29	MRSA, MRSE, MR-CoNS, MDR Pseudomonas, VRE, MDR Acinetobacter	10 yr	Two-stage	82.8%
Siddiqui et al. [25]	2013	J Arthopasty	8	MRSA	2 yr	Two-stage	88%
Mittal et al. [26]	2007	JBJS	37	MRSA, MRSE	4.25 yr	Two-stage	76%

DTT, difficult to treat; MRSA, methicillin-resistant Staph. aureus; MRSE, methicillin-resistant Staph. epidermidis; MR-CoNS, multi-resistant coagulase-negative staphylococcus; MDR, multi-drug resistant; yr, years; DAIR, debridement, antibiotics, and implant retention.

## Data Availability

The data is available from corresponding author.
